# Contextual and psychosocial factors predicting Ebola prevention behaviours using the RANAS approach to behaviour change in Guinea-Bissau

**DOI:** 10.1186/s12889-017-4360-2

**Published:** 2017-05-15

**Authors:** Anna E. Gamma, Jurgita Slekiene, Gregor von Medeazza, Fredrik Asplund, Placido Cardoso, Hans-Joachim Mosler

**Affiliations:** 1 EAWAG, Swiss Federal Institute of Aquatic Science & Technology, Environmental and Health Psychology, Überlandstrasse 133, CH-8600 Dübendorf, Switzerland; 2 UNICEF Guinea-Bissau, Apartado 464, 1034 Bissau Codex, Bissau, Guinea-Bissau; 3 INASA, Instituto Nacional de Saúde Pública, Avenida Combatente da Liberdade de Pátria, Hospital 3 de Agosto, Apartado 861, 1004 Bissau Codex, Bissau, Guinea-Bissau

**Keywords:** Ebola prevention, EVD, RANAS model, Emergencies and outbreaks, Behavioural intention, Behavioural willingness, Regression analysis, Psychosocial factors

## Abstract

**Background:**

The outbreak of the Ebola virus disease (EVD) in West Africa in December 2013 was the largest Ebola outbreak in history. This study aimed to measure the underlying contextual and psychosocial factors of intentions to perform Ebola prevention behaviours (not touching people who might be suffering from Ebola, reporting suspected cases to the National Ebola Hotline, NEH) in Guinea-Bissau. Geographical location, cross-border market activities, poor water, sanitation and hygiene (WASH) conditions, and burial practices in some communities pose a serious risk in terms of potential EVD outbreak and seriously hamper its prevention in Guinea-Bissau.

**Methods:**

In July and August 2015, quantitative data from 1369 respondents were gathered by structured face-to-face interviews. The questionnaire was based on the psychosocial factors of the RANAS (risks, attitudes, norms, abilities, and self-regulation) model. Data were analyzed by multiple linear regression analyses.

**Results:**

The most important predictors for the intention to call the NEH were believing that calling the Hotline would help the infected person, perceiving that important members from the household approve of calling the Hotline, thinking that calling the Hotline is something they should do, and believing that it is important to call the Hotline to report a suspected case. For the intention not to touch someone who might be suffering from Ebola, the most important predictors were health knowledge, the perception of risk with regard to touching a person who might be suffering from Ebola, and the belief that they were able not to touch a possibly infected person. Age in years was the only significant contextual predictor for one of the two behavioural intentions, the intention to call the Hotline. It seems that younger people are more likely to use a service like the NEH than older people.

**Conclusions:**

Strengths and gaps were identified in the study population in relation to the intention to perform prevention behaviours. These call for innovative ways of aligning existing hygiene programs with relevant psychosocial factors. This research is relevant to further outbreaks of contagious diseases as it sheds light on important aspects of the impact of public health interventions during emergencies and epidemics.

## Background

The outbreak of the Ebola virus disease (EVD) in West Africa, which started in Guinea in December 2013, was the largest and most complex Ebola outbreak in history. By the end of March 2016, 28,646 cases of Ebola virus disease were confirmed, probable, or suspected, and 11,323 deaths had been reported [1]. The most severely affected countries were Guinea, Liberia, and Sierra Leone; the virus spread across land borders from Guinea to the other two countries, with Nigeria, Senegal, Mali, Spain, Italy, and the United States of America also reporting cases.

EVD is a severe illness in humans with case fatality rates between 25% and 90% and an average fatality rate of around 50% [2]. Wild animals, such as bats and monkeys, can transmit the virus to people; it then spreads through human-to-human transmission via direct contact with the body fluids (stool, vomit, blood, urine, saliva, semen, breast milk) of infected people and by contact with surfaces or equipment contaminated by the body fluids of an infected person [3].

In July 2015, the Emergency Committee convened by the WHO (World Health Organization) Director-General recommended strengthening Guinea-Bissau’s EVD preparedness and its prevention and response capacities [3]. Guinea-Bissau was vulnerable to a potential Ebola outbreak for several reasons. Indeed, Guinea-Bissau remained at high risk throughout the regional epidemic due to its proximity to Guinea, with some cases confirmed just a few kilometers away from the border with Guinea-Bissau. Cross-border market activities, burial ceremonies and poor water, sanitation, and hygiene conditions in many communities [4] were among the main factors contributing to Guinea-Bissau’s high EVD-related vulnerability. To be prepared for the eventuality that EVD would affect the country, the government of Guinea-Bissau opened new field hospitals and arranged a procedure to evacuate suspected cases to health centers.

Since an EVD vaccine is still in the testing phase, and has been mainly used to contain flare ups in 2016 under an emergence use protocol [5], prevention behaviours play a crucial role in avoiding further transmission of the virus. The main transmission route is human-to-human contact, and preventive behaviours can avoid or reduce transmission. Underlying psychosocial factors are key aspects of such behaviours and ought thus to be taken into account.

A person suffering from Ebola needs to be treated and isolated in a health center. To enable rapid communication in a suspected case of Ebola, the Health Ministry launched the NEH. One objective of this study was to determine the strength of the intention of the population in Guinea-Bissau to use the NEH to report a suspected case of EVD.

If a person might be suffering from Ebola in the household, another very important behaviour is not to touch this person, due to the high risk of infection via direct contact with their body fluids. Taking care of a sick person is often the responsibility of close family members and puts them at high risk of being infected as well. However, not touching someone who might be suffering from Ebola could be seen as disloyal and selfish by others. The second objective of this study was therefore to reveal the psychosocial factors of the intention not to touch someone who might be suffering from Ebola. As there were no cases of EVD in Guinea-Bissau, compliance with the two prevention instructions, to report a suspected case via the NEH and not to touch someone who might be suffering from Ebola, could not be measured directly. For this reason, behavioural intention and behavioural willingness were measured.

Richards et al. [6], analyzing the social pathways for EVD in rural Sierra Leone, emphasize the role of the family as an important social factor in Ebola transmission. They found that trust was highest for the study participants in household members and extended family, and they expected to find assistance mainly within the family of the EVD case. Nevertheless, trust and respect towards authority is also high, especially for local and traditional leaders [6]. A few months after the outbreak, local chiefs, religious authorities, and local opinion leaders were included in public health interventions against EVD, since messages from these sources proved to be taken seriously by the population.

Reporting a suspected case, for instance by calling the NEH, and not touching someone who might be suffering from Ebola are prevention behaviours. When seeking to increase the population’s prevention behaviours towards EVD, it is to be acknowledged that some people have a high intention to follow instructions, while others do not. The impact of prompt human behavioural response on the infection rate was the research topic of Hu K, Bianco S, Edlund S and Kaufman J [7]. They used epidemiological modeling to quantitatively investigate the effects of behavioural changes on the transmission of EVD. In particular, the social distancing of infected individuals, which includes not touching a person who might be suffering from Ebola, could significantly reduce the transmission of the disease, mainly between infected individuals and their close social environment, such as family members, neighbours, and friends.

The purpose of this study was to identify the contextual and psychosocial factors influencing compliance with Ebola prevention instructions in a population. The underlying psychosocial factors that influence and determine behaviour can be altered with psychosocial interventions [8, 9]. Aboud FE and Singla DR [10] stress the importance of understanding the willingness and ability of a target audience to follow health messages in developing adequate behaviour change interventions. Besides this, they point out the importance of taking into account behaviour change theories and evidence from previous interventions.

Throughout the 18-month period of EVD preparedness in Guinea-Bissau, UNICEF and its partners worked in synergy with the Government of Guinea-Bissau to strengthen the country’s health system and other capacity to face a potential Ebola outbreak.

In line with the WHO’s consolidated preparedness EVD checklist [3], the priorities were to strengthen overall institutional coordination, establish a rapid response team, increase public awareness and community engagement, build infection prevention, control, and case management capacity (both at Ebola treatment centres and for safe and dignified burials), establish and strengthen epidemiological surveillance, and systematize contact tracing.

The main priorities and challenges included constantly strengthening detailed coordination amongst partners, between partners and government and within government entities; monitoring actions across the country; and supporting coordination between the health authorities of Guinea-Bissau and Guinea to increase bilateral cooperation in Ebola prevention, preparedness, and response (as well as other health issues).

Strengthening community engagement, including through dialogue with traditional and religious leaders, and linking these community structures with the national response mechanism remained amongst the most critical priorities throughout the 18-month period of EVD preparedness in Guinea-Bissau. The study presented here was very closely aligned with this cornerstone of EVD preparedness in Guinea-Bissau.

The study took place in rural, peri-urban and urban regions of Guinea-Bissau. Different promotional activities were implemented, and the NEH number was communicated to the population through a variety of channels like radio spots, leaflets, Ebola training sessions in schools and health centers. However, mobile network coverage is problematic in some areas of Guinea-Bissau.

The RANAS model, an acronym whose letters stand for risks, attitudes, norms, abilities, and self-regulation [11], was used to identify the psychosocial factors and as a theoretical background of the present study. This model is an established one for designing and evaluating behaviour change strategies in developing countries. Psychological factors proposed by major theories of behaviour change (e.g. the health belief model; the health action process approach; the theory of planned behaviour) are integrated in the RANAS model. The model also provides behaviour change techniques that tackle the factors to be changed. The RANAS model incorporates five blocks of psychological factors that have to be favorable for a new health behaviour to be assimilated: risk factors, attitudinal factors, norm factors, ability factors, and self-regulation factors. Risk factors represent the understanding and awareness of an individual about a health risk and the perceived consequences of a disease [11]. Attitudinal factors include beliefs about the costs and benefits of a certain behaviour. Normative factors are convictions about the behaviour performance of the social environment and what the social environment thinks about a certain behaviour. Ability factors include the perceptions of an individual about their personal ability to execute the behaviour. Finally, self-regulation factors are those that are responsible for the continuation and maintenance of the behaviour. First, the factors that influence the target behaviour in a given population should be determined. Then, specific interventions can be chosen from the behavioural interventions provided by the RANAS model to tackle these influencing factors.

But behaviours and behavioural intentions are not determined only by psychosocial factors. Dreibelbis et al. [12] concluded from their systematic review of behavioural models that aspects of the physical and natural environment are often underrepresented in WASH-related behaviour change theories. Several studies have demonstrated the relationship between contextual factors and WASH behaviours [13–15]. The RANAS model considers social, physical and personal contextual factors [16]. Demographic factors like age, sex and education belong to the personal context, whereas wealth belongs to the social context. We do not differ between these categories when we talk about contextual factors in the following.

Two research questions are addressed in this paper: Which contextual factors predict the intention to follow Ebola prevention instructions? Which are the crucial psychosocial factors for the intention to follow Ebola prevention instructions? The outcomes of this study can help to design efficient behaviour change interventions and to address the behavioural barriers in this population.

## Methods

### Procedure

The cross-sectional data collection took place in July and August 2015 and was accomplished in a paper and pencil format. The study area consisted of all nine regions in Guinea-Bissau: Tombali, Quinara, Oio, Biombo, Bijagos, Bafatá, Gabú, Cacheu, and Bissau. Villages were randomly selected in all regions. The sample sizes in the different regions were determined relative to the population sizes of the regions.

A team of 20 national and local health sector employees carried out structured face-to-face interviews in randomly selected households using the random-route method [17]. The interviewers were sent to randomly selected intersections; from there, they selected the households according to the protocol defined by Hoffmeyer-Zlotnik [17].

Each interview took around one hour. Only one person per household was interviewed. Most of the interviews were carried out in Creole, while a small minority were in Bijago, both local languages. Five supervisors coordinated and monitored the interviews and accompanied the data collectors in the field during the data collection. Prior to the data collection, the interviewers attended a seven-day intensive training programme, during which they learned about the study’s methodology, its goals, and the theoretical background of the study questionnaire. The data collectors learned how to fill the questionnaire and practised the translation of the questions into the local languages. The village chiefs were informed about the study to be conducted. The study also obtained the approval of the Ethical Committee of the Ministry of Public Health Guinea-Bissau. All study participants provided their written informed consent. One hundred and thirty (9.5%) households did not want be interviewed.

### Sample

The sample includes data from 1369 respondents. Most of the study participants (*n* = 744) were women, who in Guinea-Bissau are the primary care providers for their families, meaning that they are responsible for the care of sick family members. Men (*n* = 625) were interviewed as well, although they are normally not responsible for taking care of the sick. The aim of interviewing men was to detect differences in prevention behaviours and because they are also relevant to EVD transmission and prevention. As data collection took place in the rainy season, the villages selected had to be accessible. The data collectors reported that in some regions where not many or no Ebola messages were communicated, people did not feel comfortable talking about it. The mean age of the respondents ranged between 15 and 87 years (*M* = 38.19, *SD* = 13.79). While 34% of the sample had never attended school, 17.6% had completed primary school, 14.4% secondary school, and 26.6% high school.

A majority, 50.6%, of the participants were Muslim, 30.2% were Christians and 9.4% had traditional beliefs. The rest had another religion or no religion.

Agriculture (47.8%) was the main type of livelihood, followed by daily labor (13.5%), formal work (12.6%, formal working refers to ordinary employment arrangements), and commerce (11.2%). On average, 10 people lived in a household (*SD* = 7.22), and the mean number of children under the age of five in the study households was 1 (*SD* = 1.75).

The population of Guinea-Bissau is heterogeneous in ethnicity and religion, both between and within different regions.

### Questionnaire and measures

The questionnaire included the psychosocial factors from the RANAS model [11], the intention and willingness to follow the prevention instructions (to call the NEH, and not to touch a person who might be suffering from Ebola), socio-demographic characteristics and measures of the socio-economic status. Each psychosocial factor was covered by one or more items and scales were built whenever a factor was measured by more than one item. The applicability of the questionnaire was verified by a pre-test at the end of the interviewers’ training.

### Intention to follow prevention instructions

Because it was not possible to measure the behaviour directly, behavioural intention and behavioural willingness were used as outcome variables. Behavioural intention shows the motivation of a person and how hard a person is willing to try to perform the behaviour [18]. Behavioural willingness is defined as what an individual is willing to do under certain circumstances [19]. Intention and behavioural willingness are highly correlated; nevertheless, behavioural willingness explains additional variance in the behaviour [20]. Behavioural intention for the two prevention behaviours was operationalized by a direct question on a self-reported 5-point Likert scale from 1 (not at all) to 5 (very strongly) (see Table 1). To measure the behavioural willingness, the respondents had to imagine themselves in a given situation and to state the degree of their willingness to perform the two prevention behaviours on 5-point Likert scales from 1 (not at all willing) to 5 (very willing). The means of these items were combined for the analysis.

**Table 1:** Questions to measure the intention and behavioural willingness of the two prevention behaviours

Scale/construct	Example item	Scale (min/max)
Intention to call the National Ebola Hotline	How strongly do you intend to call the National Ebola Hotline if you had a person with suspected Ebola in your household?	1–5
	Now we would like to ask you to imagine yourself in a certain situation. Suppose you were the whole day at the market, to sell vegetables. At the end of the day, you go home and you find a member of your family who is vomiting and the vomit contains blood, which could be a symptom for Ebola. In those circumstances, how willing would you be to do the following?	1–5
	– To call the National Ebola Hotline and report the suspected Ebola case in your household.	
Intention not to touch someone who might be suffering from Ebola	How strongly do you intend to avoid to touch somebody who could be suffering from Ebola in your household?	1–5
	“Now we would like to ask you to imagine yourself in a certain situation. Suppose you were the whole day at the market, to sell vegetables. At the end of the day, you go home and you find a member of your family who is vomiting and the vomit contains blood, which could be a symptom for Ebola. In those circumstances, how willing would you be to the following?	1–5
	– NOT to touch the sick person, thus reducing the risk of contracting Ebola.	

*Notes.* 1 indicates the lowest value on the scale, and 5 represents the highest value on the scale (1 = not at all, 2 = a little, 3 = medium, 4 = strongly, 5 = very strongly)

### Contextual factors

Six contextual factors were included in the analysis (see Table 2). To measure wealth, an index was compiled of self-reported ownership of different goods: whether the respondent owned a computer, fridge, radio, or television, had electricity in their house, owned a clock, bicycle, car, carriage, mobile phone, motorbike or scooter, and/or boat with motor. The coded responses were summed and divided by 12, which is the maximum obtainable score. The final scale ranges from 0 (no wealth) to 1 (high level of wealth). Literacy was operationalized on a 3-point Likert scale from 1 (can neither read nor write) to 3 (can read and write). For the intention to call the NEH, we asked whether respondents had a mobile phone in the household.

**Table 2:** Contextual factors and characteristics of the study participants

Variables	Scale	*n*	M	SD
Age in years		1313	38.19	13.79
Household size		1365	10.91	7.22
Wealth	0–1	1362	.33	.20
		*n*	%	
Gender (% men)	0	626	45.7	
Gender (% women)	1	743	54.3	
Owning a mobile phone		1370	76.8	
Literacy (% can neither read nor write)		493	36.5	
Literacy (% can read only)		19	1.4	
Literacy (% can read and write)		839	62.1	

*Notes.* *M* mean, *SD* standard deviation

### Psychosocial factors

To measure the psychosocial factors, one or more items were used, and their means formed the factors used in the statistical analysis. Most of the answers were given on a 5-point Likert scale, and all items were unipolar (see Tables 3 and 4).

**Table 3:** Questions to measure the psychosocial factors for the intention to call the NEH

Scale/construct		Example item	Scale (min/max)
Risk factors	Vulnerability	How high do you feel is the risk that you get Ebola?	1–5
	Severity	Imagine that you contracted Ebola, how severe would be the impact on… … your life in general?	1–5
	Health knowledge	Can people transfer Ebola to others immediately after being infected?	Multiple choice answers: 0 = answer was wrong, 1 = answer was right
Attitude factor	Response belief	How certain are you that calling the Hotline will help you or a person who might be suffering from Ebola?	1–5
Norm factors	Others’ behaviour household	How many people of your household would call the Hotline if there was be a person who might be suffering from Ebola in your household?	1–5
	Others’ behaviour village	How many people of your village would call the Hotline if there was be a person who might be suffering from Ebola in the same household?	1–5
	Others’ (dis)approval household	People who are important to you, like your family members, how much do they approve or not that you would call the Hotline if there would be a person who might be suffering from Ebola in your household?	1–5
	Others’ (dis)approval village	People who are important in the village like an Imam or a Marabout, do they approve if you would call the Hotline and report the suspected Ebola case or not?	1–5
	Personal importance	How strongly do you feel a personal obligation to yourself to call the Hotline if there would be a person who might be suffering from Ebola in your household?	1–5
Ability factors	Action knowledge	Can you tell me the number of the National Ebola Hotline?	0–1
	Confidence in performance	How difficult would it be to call the Hotline and report the suspected Ebola case in your household?	1–5
Self-regulation factor	Commitment	How important is it for you to to call the Hotline and report the suspected Ebola case in your household?	1–5

*Notes.* 1 indicates the lowest value on the scale, and 5 represents the highest value on the scale (1 = not at all, 2 = a little, 3 = medium, 4 = strongly, 5 = very strongly)

**Table 4:** Questions to measure the psychosocial factors for the intention not to touch someone who might be suffering from Ebola

Scale/construct		Example item	Scale (min/max)
Risk factors	Vulnerability	How high do you feel is the risk that you get Ebola?	1–5
	Severity	Imagine that you contracted Ebola, how severe would be the impact on… … your life in general?	1–5
	Health knowledge	Can people transfer Ebola to others immediately after being infected?	Multiple choice answers: 0 = answer was wrong, 1 = answer was right
	Risk touching	How high do you think is the risk that you contract Ebola, if you touch a person who is suffering from Ebola?	1–5
Attitude factor	Response belief	How certain are you that not touching a sick person prevents you from contracting Ebola?	1–5
Self-regulation factor	Control not to touch	How much control do you have over whether you don’t touch a person who might be suffering from Ebola while taking care of this person at home?	1–5
Additional factor	Opinion of others	What would others think if you don’t touch a person who might be suffering from Ebola?	Open question

*Notes.* 1 indicates the lowest value on the scale, and 5 represents the highest value on the scale (1 = not at all, 2 = a little, 3 = medium, 4 = strongly, 5 = very strongly)

Health knowledge was measured with multiple-choice questions [21]. For each question, the respondent had to decide whether it was correct or not; for each correct answer, the respondent received one point, and these were finally summed. For the intention to call the NEH, action knowledge was operationalized with an open question with responses coded as 0 (doesn’t know the number of the NEH) or 1 (knows the number). For the intention not to touch someone who might be suffering from Ebola, an open question was asked to reveal how the opinion of others could influence this intention. The answers were coded into four answer categories as 1 (“they would think I am a not a nice person”), 2 (“they would think I am crazy”), 3 (“they would think I don’t want to help this person”) and 4 (“they would think I am selfish”). The four categories were integrated in the analyses as binary variables.

### Data analyses

To answer the research questions, forced-entry multiple linear regression analyses were computed. First, a regression analysis was computed to identify the relevant contextual factors. Second, another regression analysis with the relevant contextual factors and the psychosocial factors was computed. For all regression models, assumptions of no multicollinearity, linearity, independent and normally distributed errors and homoscedasticity were met. For all the other results, either frequencies or descriptive analyses were calculated. All analyses were executed with SPSS 22.

## Results

On average, the study participants stated that they had the intention to call the NEH if a person might be suffering from Ebola in the household (see Table 5). Regarding the intention not to touch a person who might be suffering from Ebola, the results showed that the respondents were quite willing not to touch someone who might be suffering from Ebola (see Table 5).

**Table 5:** Means (*M*) and standard deviations (*SD*) of the intention to call the NEH and the intention not to touch a person who might be suffering from Ebola

Dependent variables	*N*	*M*	*SD*
Calling the Hotline	1018	3.96	.77
Not touching	1092	3.69	1.04

For means and standard deviations of the psychosocial factors, see Tables 8 and 9.

Except for vulnerability and others’ (dis)approval at the village level, the means of the psychosocial factors for the intention to report a suspected Ebola case in the household to the NEH were rather high. The mean value of vulnerability indicates that the respondents estimated their risk of contracting Ebola as low to medium. Severity of EVD was perceived as high, and the health knowledge was medium to high. The attitude factor, response belief, showed that most respondents were certain that calling the Hotline would help someone who might be suffering from Ebola. On average, the study participants perceived calling the NEH as something that most people in their household would do too, but only half of the people in their village. Furthermore, respondents thought that important members from the household would approve to a medium extent if they were to call the NEH and that important people from the village would approve slightly less than to a medium extent. On average, the respondents felt personally obliged to call the NEH and to report a suspected Ebola case in the household. However, the results showed that 91.3% of the respondents could not name the number of the NEH. Means regarding the confidence in performance indicate that the respondents felt confident that they could call the NEH and did not think it to be a difficult behaviour. On average, the study participants felt committed and thought it is important to report a suspected Ebola case to the NEH.

With regard to the psychosocial factors of the intention not to touch someone who might be suffering from Ebola, the respondents stated that they were certain that not touching a sick person would help them avoid contracting Ebola. The study participants felt that it is under their control whether or not they touch a person who might be suffering from Ebola while taking care of this person at home.

### Contextual predictors of the intention to follow Ebola prevention instructions

The linear regression analysis of the intention to call the NEH with contextual factors (see Table 6) identified age in years (*β* = −.168), wealth (*β* = .118), and having a mobile phone (*β* = .125) as significant predictors. Only 7.1% of the variance could be explained by the model (see Table 6). Younger people and respondents with a higher level of wealth have a higher intention to call the NEH than others. Participants who had a mobile phone reported a greater intention to call the NEH than those who did not have a mobile phone (see Table 6).

**Table 6:** Regression analysis of the intention to call the NEH with contextual predictors

Variables	*β*
Gender	−.024
Age in years	−.168***
Household size	−.050
Literacy	.030
Wealth	.118***
Having a mobile phone	.125***

*Notes.* ***p ≤ .001. Adj. *R* ^*2*^ = .071. *N* = 1293

The linear regression analysis of the intention not to touch someone who might be suffering from Ebola with contextual factors (see Table 7) found literacy (*β* = .073) and wealth (*β* = .084) to be significant predictors. Again, the explanation of the variance was very low at 1.4% (see Table 7). Participants with higher literacy and a higher level of wealth have a higher intention than others not to touch someone who might be suffering from Ebola (see Table 7).

**Table 7:** Regression analysis of the intention not to touch someone who might be suffering from Ebola with contextual predictors

Variables	*β*
Gender	.013
Age in years	−.042
Household size	−.007
Literacy	.073*
Wealth	.084**

*Notes.* *p ≤ .05, **p ≤ .01. Adj. *R* ^*2*^ = .014. *N* = 1294

Data from men were collected in order to detect gender differences in the two behavioural intentions. The results from the two regression analyses with the contextual factors show that gender was not a significant predictor for the two behavioural intentions.

### Psychosocial predictors of the intention to follow Ebola prevention instructions

The significant contextual predictors were included in the regression analysis with the psychosocial factors from the RANAS model (see Tables 3 and 4). Eight psychosocial factors and one contextual factor contributed significantly in predicting the intention to call the NEH (see Table 8). The age in years (*β* = −.090), severity (*β* = .108), health knowledge (*β* = .095), and response belief (*β* = .137) predicted the intention to report a suspected Ebola case to the NEH. Three of the norm factors were positively and significantly related to a higher intention to report a suspected Ebola case to the NEH: Others’ behaviour household (*β* = .075), meaning that the respondents perceived that many others from their household would call the NEH as well; others’ (dis)approval in the household (*β* = .126), meaning that important members from the household approve of calling the NEH; and personal importance (*β* = .204), meaning that the respondents believe that calling the NEH is something they should do. Furthermore, confidence in performance (*β* = .073) and commitment (*β* = .162) correlated with a higher intention to call the NEH. Together, the factors explained 46.2% of the variance of the intention to call the NEH.

**Table 8:** Regression analysis of RANAS psychosocial determinants explaining the intention to call the NEH and reporting a suspected Ebola case

Factor group	Contextual or psychosocial determinants	M (SD)	*β*
Context	Age in years	37.50 (13.90)	−.090***
	Wealth	.31 (.20)	.014
	Having a mobile phone		.035
Risk factors	Vulnerability	2.47 (1.34)	.017
	Severity	4.46 (.68)	.108***
	Health knowledge	19.11 (4.88)	.095***
Attitude factor	Response belief	4.11 (0.81)	.137***
Norm factors	Others’ behaviour household	3.81 (1.13)	.075*
	Others’ behaviour village	3.17 (1.14)	−.024
	Others’ (dis)approval household	3.26 (.82)	.126***
	Others’ (dis)approval village	2.84 (1.12)	−.027
	Personal importance	4.06 (.73)	.204***
Ability factors	Action knowledge (Hotline number)	*n. a.*	.020
	Confidence in performance	3.98 (1.01)	.073**
Self-regulation factors	Commitment	4.15 (.69)	.162***

*Notes.* *p ≤ .05, **p ≤ .01, ***p ≤ .001. Adjusted *R* ^*2*^ = .462. *N* = 979

Seven psychosocial factors significantly predicted the intention not to touch someone who might be suffering from Ebola (see Table 9). Again, severity (*β* = .124) and health knowledge (*β* = .132) were psychosocial determinants of the intention not to touch someone who might be suffering from Ebola. Risk touching, meaning people think that there is a risk of contracting Ebola by touching a person who might be suffering from it (*β* = .210) and response belief (*β* = .121), meaning people are certain that not touching a sick person prevents them from contracting Ebola, were significant predictors of the intention not to touch someone who might be suffering from Ebola. The factors control not to touch (*β* = .132) and two of the opinions of others were found to be significant predictors of the intention not to touch someone who might be suffering from Ebola: people who think that others would see them as not a nice person (*β* = −.216) and as a person who does not want to help (*β* = −.067) have a lower intention not to touch than others. The model could explain 27.5% of the variance in the intention not to touch someone who might be suffering from Ebola.

**Table 9:** Regression analysis of RANAS psychosocial determinants explaining the intention not to touch someone who might be suffering from Ebola

Factor group	Contextual or psychosocial determinants	M (SD)	*β*
Context	Literacy		.011
	Wealth	.32 (.19)	.037
Risk factors	Vulnerability	2.38 (1.35)	−.050
	Severity	4.51 (0.65)	.124***
	Health knowledge	19.55 (4.65)	.132***
	Risk touching	4.08 (0.91)	.210***
Attitude factor	Response belief	4.20 (0.78)	.121**
Self-regulation factors	Control not to touch	4.07 (0.79)	.132***
Additional factors	1 Not a nice person		−.216***
	2 A crazy person		−.010
	3 Not a helping person		−.067*
	4 A selfish person		−.040

*Notes.* *p ≤ .05, **p ≤ .01, ***p ≤ .001. Adjusted *R* ^*2*^ = .275. *N* = 1075

## Discussion

This study aimed to determine contextual and psychosocial factors in Guinea-Bissau which predict the intention to call the NEH and the intention not to touch someone who might be suffering from Ebola. There are other studies, which investigated Ebola risk perceptions, Ebola knowledge and the prevention of the Ebola virus during the last Ebola outbreak in West Africa [22–26]. However, the populations of these studies were objectively at lower risk of contracting Ebola than the population assessed in this study.

The study participants stated that they were willing to call the NEH if they suspected a case in the household and that they were quite willing not to touch someone if that person might be suffering from Ebola. A study about Ebola risk perceptions in Germany [22] also found that most of their study participants would change their behaviour in order to prevent an outbreak of the Ebola virus. The majority of the respondents in this study has access to a mobile phone in their household. In general, the contextual factors did not explain much of the variance in the intention to follow the two prevention instructions. Wealth was the only contextual factor, which significantly predicted both intentions, although only in the regression model with the contextual factors alone. The effect of wealth was mediated through one or more of the psychosocial factors, indicating that respondents’ risk perceptions, attitudes, beliefs, abilities and self-regulation fully explained the effect of household wealth on the two behavioural intentions. The age in years was a significant predictor for the intention to call the NEH, and it remained significant in the regression model with the psychosocial factors. It seems that younger people are more likely to use a service like the NEH than older people and that the psychosocial factors did not wholly explain the effect of age on the intention to call the NEH. A recent study from New Zealand about public perceptions and knowledge of the Ebola virus found that age, sex and education were significant predictors for the variance in the number of named protective behaviours [23]. A younger age was significantly associated with a larger number of protective behaviours and a higher willingness to vaccinate. Having access to a mobile phone was only relevant in the regression model of the contextual factors predicting the intention to call the NEH, but not in combination with the psychosocial factors. The same was the case for literacy and the prediction of the intention not to touch someone who might be suffering from Ebola; this was no longer relevant when combined with the psychosocial factors.

In general, the RANAS model was able to explain the intention to follow the two Ebola prevention behaviours well. Nevertheless, there is one caveat: Even though we included a large number of covariates in the models and we were testing multiple hypotheses, we did not include any corrections for multiple comparisons in the analysis.

Factors from all five factor blocks of the RANAS model, namely risks, attitudes, norms, abilities, and self-regulation, were found to be underlying psychosocial factors for the intention to call the NEH. We revealed that the most important predictors of this intention were the following four: believing that calling the NEH will help the infected person (Response belief), perceiving that important members from the household approve of calling the NEH (Others’ (dis)approval household), that the respondents think calling the NEH is something they should do (Personal importance) and the belief that it is important to call the NEH and to report a suspected case (Commitment).

Attitude factors like response belief or outcome expectations are important determinants for behaviours in various theories in the field of social and health psychology, such as the social cognitive theory [27], the health belief model [28]and the health action process approach [29]. The importance of being confident about the ability of their government to control infectious diseases was as well found in the study from Kelly et al. [25] about perceptions and plans for the prevention of Ebola in US during the outbreak in West Africa. Normative beliefs, such as the perception of what others are doing, the perceived approval of important others in the social environment, and the belief of what should personally be done, have been important influencing factors in several handwashing studies [30–32]. Others’ (dis)approval has also been found to explain the increase in the consumption of deep-tube-well arsenic-free water in Bangladesh [33]. In our study, the perception of what others in the household might do and whether others in the household approve of calling the NEH were underlying psychosocial factors, but the normative beliefs of what people in the village would do (Others’ behaviour village) or whether they approve of calling the NEH (Others’ (dis)approval household), were not significant predictors. This might be because calling the NEH is a behaviour that is not shown to people outside the household. However, as Richards et al. [6] found, trust towards local and traditional leaders is high. Personal importance has also been found to be a predictor of habitual cleaning of household drinking water storage containers with soap and water [15] and of cleaning intentions for shared toilets in slums in Kampala [34].

Commitment strength has been found to be an important predictor for various WASH behaviours in developing countries: for choosing safe water options in Bangladesh [35], for habitual latrine cleaning in rural Burundi [36], and for handwashing with soap and water in Ethiopia and Haiti [30, 37].

Factors from the risk, attitude and self-regulation blocks of the RANAS model were found to influence the intention not to touch someone who might be suffering from Ebola. This behavioural intention was more difficult to measure, as it should not be performed; it is thus also more difficult to avoid its unintentional promotion during the study interviews. In order to avoid talking about this behaviour for a long time, we asked only a few questions about it. For this reason, not all the factor blocks of the RANAS model could be covered.

For this intention, the most important predictors were health knowledge, risk perception with regard to touching a person who might be suffering from Ebola and the belief in being able not to touch a possibly infected person (Control not to touch, Confidence in performance). The finding that a higher knowledge is significantly associated with a higher intention to perform Ebola prevention behaviours is consistent with a study from US [24], which showed that more knowledgeable respondents were more likely to believe that preventive actions will help against contracting Ebola.

Confidence in performance, which predicted both intentions to follow Ebola prevention behaviours, has also been found to be a predictor of handwashing behaviour in Haiti [38] and the use of arsenic-safe water options in Bangladesh [35]. Self-efficacy is a key factor of behaviour and affects all other psychosocial factors, according to Bandura [27].

Additional, but weaker, predictors were the perceived severity of Ebola (for both intentions) and health knowledge (for the intention to call the NEH). Health knowledge is seen as a precondition for change in Bandura’s Social Cognitive Theory [27]. However, risk perceptions and factual knowledge are only seen as secondary in major behaviour change theories [39]. Perceived severity only motivated handwashing in the case of epidemics such as cholera [31]. This finding is in line with the present study, in which severity is a motivator for the behavioural intentions to follow two prevention behaviours during an Ebola outbreak in the region.

### Implications for practice

The underlying psychosocial factors reveal that the intention to call the NEH can be increased by possible pathways to improving outcomes, for instance by a normative behaviour change technique like providing a positive group identity [16]. People who are already committed to calling the NEH will be described in an attractive way, for instance as modern, in order to increase the attractiveness of the behaviour itself. A possibility to increase the commitment are public pledges made by a number of people in public places (streets, markets, etc.) and, for instance, communicated in a radio advert. They can be interviewed and all of them can also remind the listener what the number of the NEH is. They say that they would call the Hotline if there was a suspected Ebola case in their household and that they know calling the NEH will help the affected person. This could increase the commitment of others to calling the hotline and strengthens using this service as a social norm.

A future prevention campaign to increase the intention not to touch someone who might be suffering from Ebola should focus on people’s knowledge about EVD, their risk perception about touching a person who might be suffering from Ebola, and their confidence in being able not to touch someone who might be suffering from Ebola. Contzen & Mosler [16, 37] propose enhancing people’s health knowledge by presenting facts and scenarios about the possibilities of contracting a certain disease and about the relationship between a certain behaviour and the disease by showing how situations in the everyday life of the participant can lead to the disease. The perception of risk in touching someone who might be suffering from Ebola could be tackled by informing people about personal risk and by assessing it in such a way that people understand that their health is at risk, and that even other people in the family may be put at risk by an individual’s behaviour. A range of behaviour change techniques could be used to boost people’s confidence and enable them not to touch someone who might be suffering from Ebola, for instance, by encouraging participants to seek practical or emotional support from relatives, friends, or others and by demonstrating how to react if someone may be suffering from Ebola. Another way to enhance confidence could be through demonstrating and modelling the behaviour and its consequences in everyday life, for example through a theatre play, showing that not touching a person who might be infected will protect the rest of the family from Ebola. Reasons for still touching someone even if he or she show symptoms of Ebola include the fear that others would think the respondent a bad person if he or she did not touch a suspected Ebola case and that others would think the respondent does not want to help a sick person. This is a critical barrier for a proper prevention behaviour in an outbreak of EVD or other highly contagious diseases and would need to be taken into account when designing an EVD preparedness campaign.

## Conclusions

In order to determine the most important factors that influence the intention to follow Ebola prevention behaviours, we considered both psychosocial and contextual factors. For behaviours such as reporting a suspected Ebola case to the NEH, campaign designers need to know about the availability of telephonic coverage in the country, and campaigns should also be adapted to illiterate people.

Although the perceived severity of the disease and health knowledge were predictors for the intention to follow the Ebola prevention behaviours, some other predictors, such as response belief, normative beliefs, commitment, and confidence in performance, were even more important in predicting behavioural intention. Many promotion activities focus primarily on disseminating knowledge about the risks and benefits of hygiene practices. If raising knowledge about the dangers of a disease has a relatively small effect on people’s behaviour and behavioural intentions, aid providers may need to adapt their messages to include other drivers of behaviours and intentions in their interventions.

The most recent EVD outbreak is over, but this research can be used for further outbreaks of contagious diseases, including recurrent endemic cholera bouts or the emerging Zika threat in Guinea-Bissau and elsewhere, as the results of the study presented in this paper shed light on important aspects of the impact of public health activities, especially during emergencies. A study about Ebola knowledge in Israel concluded that the greatest challenges that organizations face is to provide comprehensive information that empowers the target population to make fact-based decisions about health and reflects uncertainty [26]. Ultimately, the regional EVD outbreak ought to be used as an opportunity to channel the efforts deployed in EVD preparedness in Guinea-Bissau towards the strengthening of its public health system.

## Abbreviations

EVD: Ebola virus disease
NEH: National Ebola Hotline
RANAS: risks, attitudes, norms, abilities, and self-regulation
WASH: Water, sanitation and hygiene
WHO: World Health Organization
